# The cost of caring: understanding occupational stress among nurses at Mulago National Referral Hospital

**DOI:** 10.3389/fpubh.2026.1748547

**Published:** 2026-04-14

**Authors:** Edward Tumusiime, Evas Atuhaire, Eliot Namanya, Abraham Muhwezi, Steward Mudenda, Ronald Ouma Omolo, Nathan Mugenyi

**Affiliations:** 1 Department of Nursing, Faculty of Health Sciences, Victoria University, Kampala, Uganda; 2 Faculty of Health Sciences, Uganda Martyrs University, Kampala, Uganda; 3 Department of Community Health, Faculty of Medicine, Mbarara University of Science and Technology, Mbarara, Uganda; 4 Department of Pharmacy, School of Health Sciences, University of Zambia, Lusaka, Zambia; 5 Department of Physiology, Faculty of Medicine, Mbarara University of Science and Technology, Mbarara, Uganda

**Keywords:** Job Demands-Resources-Support (JDRS), Mulago National Referral Hospital, nurses, occupational stress, Uganda

## Abstract

**Background:**

Occupational stress is prevalent among nurses, particularly in resource-constrained settings, where high patient volumes, limited institutional support, and societal pressures exacerbate strain. In Uganda, nurses at tertiary referral hospitals face compounded challenges, yet empirical evidence on multilevel determinants of stress is limited. This study applied the Job Demands-Resources-Support (JDRS) framework to examine health system, patient-related, and community-level factors influencing occupational stress among nurses at Mulago National Referral Hospital (MNRH).

**Methods:**

A facility-based cross-sectional study was conducted from April to June 2024, enrolling 231 nurses through availability sampling. Occupational stress was measured using a self-reported binary indicator, and explanatory variables captured job demands, control, and support per the Job Demand-Control-Support (JDCS) framework. Descriptive statistics summarized variables, while bivariate and domain-specific multivariable logistic regression analyses were used to examine adjusted associations with occupational stress within health system, patient-related, and community-level domains.

**Results:**

Occupational stress prevalence was 64.5%. In domain-specific multivariable analyses, caregiver-related challenges were associated with higher odds of stress (aOR = 1.3, 95% CI: 1.02–1.57), while participation in continuous professional development-based programs (aOR = 0.3, 95% CI: 0.12–0.98) and lack of hospital accommodation (aOR = 0.2, 95% CI: 0.04–0.80) were associated with lower odds. Counterintuitive inverse associations were also observed for inadequate staffing and exposure to aggressive patient behavior, likely reflecting adaptation, selection effects, or residual confounding. Gender-related stressors were similarly inversely associated with stress (aOR = 0.7, 95% CI: 0.52–0.88).

**Conclusion:**

Nurses at MNRH experience high occupational stress driven by complex interactions of job demands, limited resources, and societal pressures. Effective stress mitigation requires integrated interventions addressing structural capacity, professional development, and social valuation of nursing. Future research should employ longitudinal and mixed-methods designs to disentangle the dynamics of occupational stress in high-demand, resource-limited environments.

## 1 Introduction

Occupational stress is a prevalent and multifaceted concern in healthcare systems worldwide, especially among nurses, who are the largest proportion of frontline healthcare providers and bear disproportionate burden due to the inherently demanding nature of their work. Stress, conceptualized as a physiological and psychological response to demanding or threatening circumstances, plays a dual role as it can be adaptive in moderation but becomes detrimental when chronic or excessive, ultimately impairing wellbeing and job performance (1, 2). In the nursing profession, persistent exposure to high workload, long working hours, emotional strain, and inadequate institutional support significantly contributes to burnout, diminished job satisfaction, and compromised quality of patient care (3, 4).

The Job Demands-Resources-Support (JDRS) theoretical framework offers a robust lens for analyzing occupational stress by positing that employee wellbeing is determined by the balance between job demands and the resources and support available to meet those demands. According to this model, job demands including excessive workload, time pressure, and emotionally demanding patient interactions, are associated with sustained effort and psychological costs, leading to stress and burnout when excessive. In contrast, job resources such as autonomy, social support, adequate staffing, and opportunities for professional development, promote work engagement and buffer the negative effects of high demands (5–7). Moreover, support mechanisms, whether managerial, peer, or organizational, are essential in mitigating the impact of job stressors and enhancing nurse resilience and motivation, especially in high-pressure clinical settings (8, 9). The JDRS model thus provides a theoretically grounded structure for examining how health system factors, patient-related complexities, and community influences interact to shape nurses' stress experiences and outcomes.

Globally, research consistently shows nurses sustain some of the highest levels of work-related stress among healthcare professionals (10). Regional studies estimate burnout prevalence ranging from approximately 35%−54% in high-income countries like the United States, linked to excessive workload and administrative burdens (11, 12), to between 40 and 80% in sub-Saharan Africa, where resource limitations and staff shortages compound demands on nurses' physical and emotional capacities (13, 14). In low and middle-income countries (LMICs), such as in parts of East Africa, high patient volumes, limited institutional support, and lack of recognition further exacerbate stress among nurses, negatively affecting workforce sustainability and care delivery (15, 16).

In Uganda, occupational stress among nurses presents a critical health system issue. Approximately four in ten nurses report high stress or burnout levels, a prevalence that escalated during the COVID-19 pandemic (17). At Mulago National Referral Hospital (MNRH), Uganda's largest tertiary healthcare facility and the operational core of the national health system, chronic understaffing, inadequate supply chains, limited accommodation, and prolonged work hours are consistently identified as prominent system-level stressors. These challenges coexist with high patient volumes, complex comorbidities, aggressive patient behaviors, and communication barriers rooted in diverse sociocultural contexts, and these factors intensify cognitive and emotional demands on nurses. Additionally, community-level and societal influences, such as gendered professional stereotypes and limited public appreciation of nursing, further compound stress by undermining social support and professional recognition (18–21).

Persistent occupational stress among nurses at MNRH and similar settings has far-reaching consequences for health system performance. Stress and burnout among nurses are linked to increased absenteeism, higher turnover intentions, reduced productivity, compromised patient safety, and poorer patient satisfaction, all of which undermine quality of care and system sustainability (22–24). For example, studies show that high burnout and unmet work demands among nurses correlate with diminished adherence to safety protocols and increased risk of adverse patient outcomes, highlighting the intersection between staff wellbeing and the quality of care delivered (25).

Despite the recognized burden of stress among nurses globally and in Uganda, there is a gap in empirical evidence that systematically investigates how health system, patient-related, and community-level stressors interact to influence occupational stress within major referral hospitals, the nexus where high acuity, capacity constraints, and institutional complexity converge. Previous studies in Uganda have largely focused on specific contexts, such as pandemic responses or district-level facilities, without applying a comprehensive theoretical framework that unpacks the multidimensional determinants of nurse stress in a high-pressure national referral setting.

While the study is theoretically grounded in the JDRS model, measurement of key constructs draws on the Job Demand-Control-Support (JDCS) framework (26, 27) to ensure alignment with established occupational stress instruments and facilitate operationalization of autonomy and support. Accordingly, this study applies the JDRS model to assess the determinants of occupational stress among nurses at Mulago National Referral Hospital, Kampala District. Specifically, it examines how health system factors, patient-related factors, and community influences contribute to stress experiences, and how resources and support systems may buffer these relationships among nurses. Findings from this study aim to inform institutional policies, strengthen supportive work environments, and enhance strategies to improve nurse wellbeing and healthcare delivery outcomes in Uganda.

## 2 Materials and methods

### 2.1 Study design and theoretical framework

This study employed a facility-based quantitative cross-sectional design conducted from April to June 2024, at Mulago National Referral Hospital, Kampala, Uganda. The design was appropriate for estimating the prevalence of occupational stress and examining associations between stress and multiple explanatory factors at a single point in time. The study was guided by the JDRS model (5–7), which conceptualizes occupational stress as a function of the balance between: (1) job demands (e.g., workload, patient complexity, emotional labor), (2) job resources (e.g., autonomy, availability of equipment, staffing adequacy), and (3) support mechanisms (e.g., managerial support, professional development opportunities, community recognition). While the overarching theoretical framework was the JDRS model, questionnaire measurement followed the JDCS (26, 27) structure to operationalize autonomy and support using established occupational stress constructs.

### 2.2 Study setting

The study was conducted at Mulago National Referral Hospital (MNRH), Uganda's largest tertiary and teaching hospital and the apex of the national health system. MNRH provides specialized care and serves as a referral center for complex cases nationwide, making it a high-demand clinical environment characterized by heavy workloads, complex patient profiles, and resource constraints (28, 29).

### 2.3 Study population

The study population comprised all nurses working at MNRH at the time of data collection. Nurses across all clinical departments and shifts were eligible to participate. Registered nurses who were on active duty during the study period and provided informed consent were included in the study whereas student nurses and nurses who were unavailable due to emergency clinical duties at the time of data collection were excluded in the study.

### 2.4 Sample size determination

The sample size was calculated using Fisher's formula for single-proportion estimation and adjusted using a finite population correction, given the known population size.

Initial sample size:


$$\begin{array}{l} n_{0}=\frac{Z^{2}p\left(1-p\right)}{d^{2}} \end{array}$$

Where:

- *Z* = 1.96 (95% confidence level).
- *p* = 0.40 (estimated proportion of nurses experiencing stress in Uganda).
- *d* = 0.05 (margin of error).

$$\begin{array}{l} n_{0}=\frac{{\left(1.96\right)}^{2}\times 0.4\times 0.6\;}{0.05^{2}}=369 \end{array}$$

Finite population correction:


$$\begin{array}{l} n=\frac{n_{0}}{1+\frac{n_{0}-1}{N}}=\frac{369}{1+\frac{368}{618}}=231 \end{array}$$

Thus, a final sample size of 231 nurses was obtained.

### 2.5 Power consideration

We acknowledge that the sample size yields a borderline events-per-variable (EPV) ratio for multivariable logistic regression. This limitation is explicitly acknowledged, and model parsimony was prioritized to reduce overfitting.

### 2.6 Sampling and recruitment

An availability (non-probability) sampling approach was used. This strategy was pragmatically justified due to: shift-based work schedules, High clinical workload, and Limited feasibility of probability sampling in a high-intensity tertiary hospital setting. To enhance representativeness, Data collection spanned multiple wards and departments, nurses were recruited across day, evening, and night shifts and the data collection process occurred over 20 consecutive days to capture variation in staffing patterns.

### 2.7 Study instrument and measures

#### 2.7.1 Questionnaire development and structure

Data were collected using a semi-structured, self-administered questionnaire developed through adaptation of items from existing occupational stress and nursing workload literature. While the study is conceptually framed using the Job Demands-Resources-Support (JDRS) model, the questionnaire structure and measurement approach align with the Job Demand-Control-Support (JDCS) framework (26, 27) to facilitate assessment of control (autonomy) and support, ensuring coherence with validated occupational stress survey methodologies, while maintaining the broader JDRS theoretical lens for analysis and interpretation. The questionnaire comprised four sections which are mentioned in Table 1.

**Table 1:** Structure and sections of the questionnaire used to assess occupational stress and related factors among nurses.

Section	JDCS domain	Variables
A	Demographics	Age, sex, cadre, years of experience
B	Job demands	Workload, staffing adequacy, patient complexity, emotional demands
C	Job control	Autonomy in clinical decision-making, ability to prioritize tasks, availability of supplies
D	Support	Management support, staffing support, professional development opportunities, community recognition

#### 2.7.2 Outcome variable (occupational stress)

The primary outcome variable was occupational stress, operationalized as a binary variable (Yes/No) based on self-reported experience of work-related stress.

We acknowledge that occupational stress was measured using a single-item self-report, which limits construct depth; this is addressed as a limitation.

#### 2.7.3 Validity and reliability

Content validity was assessed through expert review by three senior nursing educators and one occupational health specialist. A Content Validity Index (CVI) of ≥0.80 was achieved for all retained items, then internal consistency for multi-item subscales was assessed using Cronbach's alpha: Job Demands scale: α = 0.78, Job Control scale: α = 0.74 and Support scale: α = 0.81. The questionnaire was pilot-tested among nurses at a comparable tertiary facility, and minor contextual modifications were made.

### 2.8 Data collection procedure

Data collection was conducted over 20 days by the principal investigator and a trained research assistant. Nurses were approached during breaks or low-intensity periods to minimize disruption to patient care. Completed questionnaires were retrieved immediately to ensure completeness and confidentiality.

### 2.9 Data management and statistical analysis

Data were coded and entered into Microsoft Excel and analyzed using IBM SPSS Statistics version 25 (IBM Corp., Armonk, NY, USA). Double-entry verification was used to minimize data entry errors. Descriptive statistics (frequencies and percentages) were used to summarize demographic characteristics and occupational stress variables aligned with the JDCS measurement structure and interpreted within the JDRS framework. Bivariate logistic regression analysis was conducted to assess the association between each independent variable and occupational stress. Variables with a *p*-value < 0.20 at the bivariate level were considered for inclusion in the multivariable analysis. Given the thematic structure of the questionnaire, and the theory-informed organization of variables within the JDRS framework, multivariable logistic regression was conducted using separate domain-specific models for health system factors, patient-related factors, and community-level factors. Accordingly, adjusted odds ratios (aORs) reported in Tables 2–4 reflect adjustment within the corresponding domain and do not represent fully adjusted estimates across all study domains.

**Table 2:** Bivariate and multivariate logistic regression analysis of health system factors associated with stress (*n* = 231).

Variable	Category	Bivariate analysis		Multivariate analysis	
		cOR (95% CI)	*p*-value	aOR (95% CI)	*p*-value
Working hours per shift	8 h (ref)	—	—	—	—
	≥12 h	1.3 (0.85–2.01)	0.217	—	—
Adequate staff per shift	Yes (ref)	—	—	—	—
	No	3.5 (1.30–9.72)	0.014^*^	0.2(0.09–0.53)	0.001^*^
Management support	Yes (ref)	—	—	—	—
	No	1.0 (0.36–2.74)	0.977	—	—
Equipment/resources	Yes (ref)	—	—	—	—
	No	2.7 (0.81–8.71)	0.107	—	—
Job satisfaction programs	Appreciation and Incentives (ref)	—	—	—	—
	CPD	4.6 (1.60–13.18)	0.005^*^	0.3 (0.12–0.98)	0.045^*^
Accommodation provided	Yes (ref)	—	—	—	—
	No	4.8 (1.02–22.92)	0.047^*^	0.2 (0.04–0.80)	0.020^*^

^*^Statistically significant, CPD, continuous professional development; cOR: crude odds ratio; aOR, adjusted odds ratio; ref, reference; CI, confidence interval.

**Table 3:** Bivariate and multivariate logistic regression analysis of patient-related factors associated with stress (*n* = 231).

Variable	Category	Bivariate analysis		Multivariate analysis	
		cOR (95% CI)	*p*-value	aOR (95% CI)	*p*-value
Aggressive patients	No (ref)	—	—	—	—
	Yes	8.6 (2.67–27.78)	< 0.001^*^	0.7 (0.58–0.83)	< 0.001^*^
Multiple comorbidities	No (ref)	—	—	—	—
	Yes	9.4 (2.47–36.10)	0.001^*^	0.6 (0.51–0.76)	< 0.001^*^
High patient load	No (ref)	—	—	—	—
	Yes	3.5 (1.13–10.66)	0.030^*^	0.9 (0.71–1.11)	0.295
Caregiver challenges	No (ref)	—	—	—	—
	Yes	0.03 (0.002–0.45)	0.012^*^	1.3 (1.02–1.57)	0.032^*^
Different patient backgrounds	No (ref)	—	—	—	—
	Yes	5.4 (1.79–16.48)	0.003^*^	0.7 (0.62–0.88)	0.001^*^

^*^Statistically significant, cOR, crude odds ratio; aOR, adjusted odds ratio; ref, reference; CI, confidence interval.

**Table 4:** Bivariate and multivariate logistic regression analysis of community-related factors associated with stress (*n* = 231).

Variable	Category	Bivariate analysis		Multivariate analysis	
		cOR (95% CI)	*p*-value	aOR (95% CI)	*p*-value
Media portrayals	Do not contribute (ref)	—	—	—	—
	Contribute	1.7 (1.13–2.67)	0.011^*^	NS	—
Societal expectations	Do not contribute (ref)	—	—	—	—
	Contribute	0.8 (0.52–1.16)	0.219	NS	—
Stigma/discrimination	No (ref)	—	—	—	—
	Yes	1.6 (0.85–2.86)	0.153	NS	—
Gender-related stressors	Not significant (ref)	—	—	—	—
	Significant	2.0 (1.14–3.79)	0.016^*^	0.7 (0.52–0.88)	0.003^*^

^*^Statistically significant, cOR, crude odds ratio; aOR, adjusted odds ratio; ref, reference; CI, confidence interval; NS, not significant at Multivariate analysis.

Within each domain-specific model, variables with bivariate *p* < 0.20 were entered, and backward likelihood ratio elimination was used while retaining theoretically important JDCS/JDRS constructs to reduce within-domain confounding.

Although a single combined multivariable model including predictors from all domains could provide estimates adjusted simultaneously for health system, patient-related, and community-level factors, the present analysis retained domain-specific models to preserve the conceptual structure of the JDRS framework and to allow interpretation of clusters of related stressors within distinct contextual levels. However, this approach does not fully adjust for potential cross-domain confounding or interactions among predictors. Consequently, the adjusted associations reported in this study should be interpreted as within-domain estimates rather than fully independent effects.

For multivariable analysis, selected multi-category exposure variables were collapsed into binary (Yes/No) indicators to ensure model stability, reduce sparse cell counts, and enhance interpretability of effect estimates, consistent with standard logistic regression practice. Highly prevalent exposures with limited between-group variability (e.g., unrealistic patient expectations) were retained for descriptive analysis but were not prioritized for multivariable modeling due to their limited discriminative power in regression analysis. Descriptive distributions of the original categories are presented separately to preserve contextual detail. Results are reported as Adjusted Odds Ratios (aORs) with 95% Confidence Intervals (CIs). Reference categories for all categorical predictors are explicitly indicated as “(ref)” in the regression tables, and all textual interpretations are aligned with the direction of the reported aORs and confidence intervals. An aOR < 1 was interpreted as an association with lower odds of reported occupational stress, while an aOR >1 indicated an association with higher odds of reported occupational stress. Because multivariable adjustment was performed within domains rather than in a single unified model, residual confounding across domains is possible and is explicitly acknowledged in the limitations.

### 2.10 Ethical considerations

Ethical approval for the study was obtained from Mulago Hospital Research Ethics Committee (Approval Number: MHREC 2731). Participation in the study was voluntary, and informed consent was obtained from all participants after explaining the purpose, procedures, and potential benefits and risks of the research. Confidentiality was maintained by ensuring that no identifying information appeared on the questionnaires, and data were securely stored and accessible only to the research team. Cultural norms, institutional regulations, and professional ethics were respected throughout the study.

## 3 Results

Unless otherwise stated, multivariable results presented in this section reflect domain-specific models (health system, patient-related, and community-level factors) and should be interpreted as within-domain adjusted associations rather than fully independent effects across all domains.

### 3.1 Demographic characteristics of nurses at Mulago National Referral Hospital

Table 5 shows a total of 231 nurses participated in the study. Nearly half of the respondents were aged 36 years and above (44.6%). The majority were female (61.5%), and most were assistant nursing officers (66.2%).

**Table 5:** Demographic characteristics of nurses at Mulago National Referral Hospital (*n* = 231).

Variable	Category	Frequency (*n*)	Percentage (%)
Age (years)	18–25	29	12.6
	26–30	42	18.2
	31–35	57	24.7
	≥36	103	44.6
Gender	Female	142	61.5
	Male	89	38.5
Cadre	Assistant nursing officer	153	66.2
	Enrolled nurse	41	17.7
	Nursing officer	37	16.0

### 3.2 Prevalence of occupational stress

Overall, 64.5% (*n* = 149) of nurses reported experiencing occupational stress, while 35.5% (*n* = 82) reported no stress.

### 3.3 Health system-related job demands, control, and support factors

Table 6 presents the distribution of health system-related job demands and resources. Overall, nurses reported a high burden of structural job demands, characterized by inadequate staffing, limited institutional support, and constrained material resources. More than four-fifths of respondents reported inadequate staffing per shift (82.3%), lack of management support (83.1%), and insufficient equipment or resources (87.4%). Nearly half of nurses (48.9%) worked shifts lasting 12 h or longer. Job resources were limited, with hospital-provided accommodation reported by fewer than one in ten nurses (8.7%), and formal job satisfaction programs largely centered on continuous professional development rather than incentive-based recognition. These distributions provide the contextual burden against which inferential associations with occupational stress were examined.

**Table 6:** Health system-related job demands, control, and support factors (*n* = 231).

Variable	Category	Frequency (*n*)	Percentage (%)
Working hours per shift	8 h	118	51.1
	12 h	113	48.9
Adequate staff per shift	Yes	41	17.7
	No	190	82.3
Adequate management support	Yes	39	16.9
	No	192	83.1
Type of support received^*^	Volunteers	26	66.7
	Cover nurses	13	33.3
Adequate equipment/resources	Yes	29	12.6
	No	202	87.4
Job satisfaction programs	Appreciation and incentives	44	19.0
	CPD	187	81.0
Hospital accommodation provided	Yes	20	8.7
	No	211	91.3

^*^Among those reporting management support; CPD, continuous professional development.

### 3.4 Bivariate and multivariate logistic regression analysis of health system factors associated with stress

Within the Health System domain (Table 2), three variables were statistically associated with occupational stress in the domain-specific multivariable model. Inadequate staffing per shift was associated with significantly lower adjusted odds of reported stress after adjustment (aOR = 0.2, 95% CI: 0.09–0.53, *p* = 0.001). Similarly, participation in continuous professional development-based job satisfaction programs was associated with lower odds of stress compared with appreciation and incentive-based programs (aOR = 0.3, 95% CI: 0.12–0.98, *p* = 0.045). Lack of hospital-provided accommodation was also associated with lower adjusted odds of reported stress (aOR = 0.2, 95% CI: 0.04–0.80, *p* = 0.020). Other health system factors, including long working hours, management support, and availability of equipment or resources, were not statistically associated with stress after adjustment within the domain-specific model. The strong inverse association between inadequate staffing and reported stress was counterintuitive and is highlighted here as a key inferential finding, warranting further exploration in the discussion. These estimates reflect adjustment within the health system domain and should be interpreted in light of potential confounding from factors in other domains.

### 3.5 Patient-related job demands influencing stress among nurses

Table 7 describes patient-related job demands experienced by nurses, reflecting the clinical care burden within the JDRS demand domain. Unrealistic patient expectations were the most commonly reported patient behavior causing stress (78.4%), followed by non-compliance (12.1%) and aggressive behavior (9.5%). The majority of nurses cared for patients with multiple comorbidities (83.1%) and reported high patient volumes as a primary source of exhaustion (86.6%). Challenges related to caregivers (91.8%), diverse patient backgrounds (85.3%), and patients with financial constraints (87.4%) were also highly prevalent. These descriptive findings provide context for the inferential analysis of patient-related predictors of occupational stress.

**Table 7:** Patient-related job demands influencing stress among nurses (*n* = 231).

Variable	Category	Frequency (*n*)	Percentage (%)
Patient behaviors causing stress	Aggressive	22	9.5
	Non-compliance	28	12.1
	Unrealistic expectations	181	78.4
Patient health conditions	Multiple comorbidities	192	83.1
	Minor illness	6	2.6
	Cognitive impairment	33	14.3
Cause of exhaustion	Many patients	200	86.6
	Very old patients	6	2.6
	Complex needs	25	10.8
Caregiver challenges	Yes	212	91.8
	No	19	8.2
Different patient backgrounds	Yes	197	85.3
	No	34	14.7
Financially constrained patients	Yes	202	87.4
	No	29	12.6

### 3.6 Bivariate and multivariate logistic regression analysis of patient-related factors associated with stress

In the multivariable analysis of patient-related job demands (Table 3), caregiver-related challenges were associated with higher odds of occupational stress in the domain-specific multivariable model, with nurses reporting caregiver challenges having higher odds of stress (aOR = 1.3, 95% CI: 1.02–1.57, *p* = 0.032). In contrast, exposure to aggressive patient behavior was associated with lower adjusted odds of reported stress after adjustment (aOR = 0.7, 95% CI: 0.58–0.83, *p* < 0.001). Similarly, caring for patients from different backgrounds was associated with lower odds of stress (aOR = 0.7, 95% CI: 0.62–0.88, *p* = 0.001) after adjustment within the domain model. Although high patient load and caring for patients with multiple comorbidities were significantly associated with stress at the bivariate level, these factors were not statistically associated with stress in the adjusted domain-specific model. The lower adjusted odds of reported stress observed for aggressive patient behavior and diverse patient backgrounds are highlighted as key inferential findings and are explored further in the discussion. These findings reflect within-domain adjustment and should be interpreted cautiously given potential residual confounding from health system and community-level factors.

### 3.7 Community-level support and stressors among nurses (JDRS support domain)

Table 8 presents the descriptive distribution of community-level support and stressors among nurses within the JDRS support domain. Although community-level factors are not explicitly represented in the JDCS model, they are conceptualized here as external sources of support or stress consistent with the broader JDRS framework. Most nurses reported moderate to high professional recognition (56.3%), while the remainder perceived low recognition (43.7%). A majority of participants perceived negative media portrayals as contributing to occupational stress (90.1%), and similarly, societal expectations were reported to contribute by 88.8% of nurses. Experiences of stigma were common, with 84.9% of nurses reporting some level of stigmatization, and gender-related stressors were frequently reported (87.0%). These descriptive results contextualize the prevalence of community-related pressures experienced by nurses, highlighting key areas for potential organizational or policy interventions.

**Table 8:** Community-level support and stressors among nurses (JDRS support domain; *n* = 231).

Variable	Category	Frequency (*n*)	Percentage (%)
Professional recognition	Highly	32	13.9
	Moderately	98	42.4
	Slightly	101	43.7
Negative media portrayal	Contribute	208	90.1
	Do not contribute	23	9.9
Societal expectations	Contribute	205	88.8
	Do not contribute	26	11.2
Experienced stigma	Yes	196	84.9
	No	35	15.1
Gender-related stressors	Significant	201	87.0
	Not significant	30	13.0

### 3.8 Bivariate and multivariate logistic regression analysis of community-related factors associated with stress

In the multivariable analysis (Table 4), gender-related stressors were significantly associated with occupational stress in the domain-specific multivariable model. Nurses reporting moderate to extreme gender-related stress had 30% lower adjusted odds of reported stress compared to those reporting slight or no gender-related stress (aOR = 0.7, 95% CI: 0.52–0.88, p = 0.003). Although media portrayals were associated with higher crude odds of stress (cOR = 1.7, 95% CI: 1.13–2.67, p = 0.011), this association was not significant after adjustment for other community-level factors. Similarly, societal expectations and experienced stigma were not statistically associated with stress after adjustment within the domain-specific model. These findings highlight the complex interplay between community-level pressures and occupational stress, consistent with the JDRS support domain framework.

This inverse association is counterintuitive and is highlighted as a key inferential finding. Possible explanations related to measurement, contextual dynamics, and unmeasured confounding are explored in the discussion. These associations represent within-domain estimates and do not reflect full adjustment across all study domains.

## 4 Discussion

This study examined occupational stress among nurses at MNRH using the JDRS framework, with measurement operationalized through the JDCS model (26, 27). The findings demonstrate a high prevalence of occupational stress and reveal a complex interplay between job demands, control, and support operating within a resource-constrained tertiary hospital environment. Importantly, while several stressors showed strong crude associations with stress, some exhibited counterintuitive inverse relationships in domain-specific multivariable models, which may reflect methodological factors such as residual confounding, suppression effects, or model instability in addition to contextual explanations.

### 4.1 Occupational stress through the JDCS lens

#### 4.1.1 The overwhelming burden of job demands

Consistent with the JDCS model, this study confirms that nurses at MNRH operate under extreme job demands, driven by high patient volumes, complex clinical presentations, and substantial emotional labor. More than two-thirds of nurses reported occupational stress, a prevalence comparable to reports from other sub-Saharan African settings (13, 15) and substantially higher than estimates from many high-income countries (11, 12). This difference reflects structural inequities in workforce density, infrastructure, and health system financing that disproportionately burden nurses in LMICs.

Patient-related demands were particularly salient. Caregiver-related challenges were associated with higher odds of reported stress in the domain-specific multivariable model, highlighting the emotional and interpersonal labor, nurses perform beyond clinical care. In overcrowded referral settings such as MNRH, caregivers often substitute for absent institutional support systems, increasing nurses' cognitive load and emotional exhaustion. Similar findings have been reported in Ethiopia and Nigeria, where caregiver expectations and involvement, significantly intensify nursing workload (30, 31).

Notably, while aggressive patient behavior, high patient load, and multiple comorbidities were strongly associated with stress at the bivariate level, these associations attenuated or reversed in adjusted models. Rather than indicating that these exposures are protective, this pattern likely reflects adaptation to chronically high demands, where stress responses plateau once a threshold of exposure is exceeded. In such environments, stress may be driven less by episodic events and more by systemic constraints, consistent with the JDCS proposition that demands alone do not determine stress outcomes. However, because adjustment was performed within domains rather than in a single unified model, cross-domain confounding may also contribute to these observed patterns.

#### 4.1.2 The critical deficit in control and support

Beyond demands, the JDCS model emphasizes the buffering role of control and support, both of which were markedly constrained in this setting. Limited autonomy, inadequate staffing support, and scarce institutional resources characterized nurses' working conditions. Although management support and equipment availability did not remain statistically associated with stress after adjustment within the domain-specific model, their near-universal inadequacy suggests insufficient variability to exert discriminative power in regression models, rather than lack of substantive importance.

Participation in continuous professional development (CPD)-based satisfaction programs was associated with lower odds of stress compared to appreciation or incentive-based programs. This finding suggests that, within highly constrained systems, skill acquisition and professional competence may provide a stronger sense of control than symbolic recognition alone. Similar patterns have been observed in LMIC settings, where professional development opportunities enhance perceived agency even in the absence of material rewards (16, 32).

The absence of hospital-provided accommodation was also associated with lower adjusted odds of stress, another counterintuitive finding. This may reflect unmeasured confounding, whereby nurses able to secure independent housing possess greater socioeconomic stability, autonomy, or social support networks. Alternatively, institutional accommodation may be allocated preferentially to junior or more heavily burdened staff, introducing selection effects. These interpretations should be considered exploratory, as the domain-specific modeling strategy does not fully adjust for factors operating across health system, patient-related, and community contexts. These findings reinforce the JDCS principle that control and support are relational and context-dependent, not uniformly protective. This pattern mirrors the paradoxical staffing finding discussed in Section 4.3, reinforcing the presence of cross-domain suppression effects rather than true protective relationships.

#### 4.1.3 Contextualizing stressors: the Ugandan JDCS profile

When situated within the global JDCS literature, the stress profile observed at MNRH reflects a high-demand, low-control, low-support configuration, a pattern strongly associated with adverse mental health outcomes (6, 27). In high-income settings, occupational stress among nurses is often driven by administrative burden, documentation pressure, and role ambiguity (33). In contrast, this study demonstrates that in Uganda, structural scarcity and patient overload dominate, while formal organizational supports remain weak.

Community-level stressors further differentiate the Ugandan context. Although not explicitly included in the JDCS model, community recognition, media portrayal, stigma, and gender norms align with the support dimension of the broader JDRS framework. Their inclusion enhances explanatory depth by capturing external social forces that shape workplace stress. Similar extensions of JDCS to include societal context have been advocated in recent occupational health literature, particularly in LMICs where professional identity is heavily socially mediated (19, 21).

Across job demand, control, and support domains, a consistent analytical pattern was observed in the domain-specific models: several high-exposure stressors demonstrated positive crude associations but attenuated or inverted after adjustment. This pattern was evident for inadequate staffing, aggressive patient behavior, and gender-related stressors. Rather than indicating true protective effects, such directionality may reflect residual confounding, suppression effects, collinearity among chronic stressors, limited between-group variability, or coefficient instability under multivariable adjustment. Within the JDCS framework, this phenomenon is theoretically plausible in settings where demands, control, and support are simultaneously constrained; however, because the models were estimated separately by domain, these findings should be interpreted with particular caution and not as fully independent effects.

When demands, control, and support are simultaneously constrained, individual stressors may lose marginal explanatory power once broader structural conditions are accounted for. In such contexts, stress becomes less responsive to single exposures and more reflective of cumulative systemic strain. Recognizing this pattern is critical to avoiding misinterpretation of adjusted estimates as substantively protective.

### 4.2 Deep comparative analysis: LMIC vs. high-income contexts

The paradoxical attenuation of several stressors in multivariable models contrasts with findings from high-income countries, where specific exposures (e.g., patient aggression) often retain strong adjusted associations (34). In LMIC settings, chronic exposure to multiple simultaneous stressors may normalize extreme working conditions, reducing perceived marginal stress attributable to individual factors. This phenomenon aligns with theories of stress habituation and cognitive adaptation, where persistent adversity reshapes baseline expectations and coping thresholds (1).

Additionally, limited availability of alternative employment and strong professional identity may compel nurses to cognitively reframe stressors as inherent to the role, particularly in public referral hospitals. These contextual dynamics underscore why direct transposition of findings from high-income contexts to LMICs is often misleading without theoretical grounding.

### 4.3 Dedicated analysis of the anomalous finding: inadequate staffing

The most striking and methodologically important finding in this study is the strong inverse adjusted association between inadequate staffing and reported occupational stress. From a methodological perspective, this inverse association may reflect residual confounding, coefficient instability due to limited events-per-variable ratios, or the domain-specific modeling strategy, which does not fully adjust for predictors across domains. These statistical factors may contribute to direction reversal under multivariable adjustment. This result contradicts extensive global evidence linking understaffing to burnout and psychological distress (22, 35) and must be interpreted cautiously.

Several plausible explanations merit consideration. First, measurement limitations may have contributed; staffing adequacy was assessed as a binary perception rather than an objective ratio, potentially conflating chronic normalization with actual adequacy. Second, adaptation to severe understaffing may lead nurses to recalibrate stress expectations, reporting stress primarily in relation to other psychosocial factors rather than staffing itself. Third, residual confounding is likely, as more experienced or resilient nurses may be preferentially assigned to severely understaffed units, while novice staff receive additional support. Rather than dismissing this finding, it should be viewed as an indicator of complex stress dynamics in chronically constrained systems. Future studies employing longitudinal designs, objective staffing metrics, and qualitative inquiry are essential to disentangle these mechanisms.

### 4.4 Implications for practice and policy

The findings of this study provide actionable insights for multilevel interventions aimed at reducing occupational stress among nurses working in high-demand, resource-limited settings. The recommended actions span immediate managerial practices, hospital-wide institutional policies, and broader national-level advocacy, and are informed by the observed associations identified in the study.

At the unit level, immediate managerial actions should focus on addressing patient-related stressors and strengthening professional capacity. Mandatory training in de-escalation, conflict resolution, and effective communication should be implemented to support nurses in managing aggressive patient behaviors and caregiver-related challenges. In addition, structured support mechanisms, such as temporary relief staffing or strategic shift rotations, are necessary for nurses managing high patient loads or complex comorbid cases. Professional development should also be prioritized through the promotion of continuing professional development (CPD) programs that emphasize skill acquisition and career advancement, as these were associated with lower reported odds of occupational stress. Establishing mentorship and peer-support systems can further enhance nurses' perceived control, resilience, and coping capacity within clinical units.

At the institutional level, hospital-wide policies should address staffing adequacy, accommodation challenges, workplace culture, and occupational wellbeing. Regular staffing audits are recommended to ensure that workforce allocation aligns with workload demands. Hospitals should also explore housing support initiatives, including staff housing funds or partnerships with housing providers, particularly for junior nurses and those working in high-demand units. To improve job satisfaction, institutions should expand recognition strategies beyond professional development to include tangible incentives such as awards and performance-based acknowledgments. Strengthening participatory decision-making and enhancing managerial responsiveness are equally important for improving autonomy, support, and trust within the workplace. Furthermore, hospitals should introduce accessible occupational health services, including counseling, stress management programs, and employee assistance initiatives, to address nurses' mental health and wellbeing.

At the national and policy level, advocacy efforts should focus on improving societal perceptions of nursing, addressing gender-related stressors, and aligning health workforce policies with the realities of practice. Public awareness campaigns, developed in collaboration with media organizations and professional associations, can help counter negative portrayals of nursing and promote societal appreciation of the profession. Gender-sensitive policy interventions are also essential, including mentorship programs for female nurses and workplace policies that address gender-specific stressors. In addition, national health workforce policies should prioritize the establishment of staffing standards, CPD-linked career incentives, and housing support schemes to enhance nurse retention and workforce sustainability, particularly in LMICs.

Overall, occupational stress among nurses arises from interacting factors related to job demands, control, social support, and broader societal contexts. Effective and sustainable stress-reduction strategies must therefore adopt an integrated, multilevel approach that combines individual skills development, organizational policy reforms, and national advocacy efforts to meaningfully improve nurses' working conditions and wellbeing.

### 4.5 Strengths and limitations

This study has several notable strengths. It is grounded in established occupational stress theory and applies the JDRS framework, operationalized through the JDCS model, providing a theoretically robust lens for interpreting multilevel determinants of occupational stress. The focus on Uganda's largest tertiary referral hospital enhances the relevance of the findings to high-demand, resource-constrained health systems in LMICs. In addition, the integration of health system, patient-related, and community-level factors offers a comprehensive understanding of the contextual drivers of stress among nurses.

Despite these strengths, several limitations should be acknowledged. First, the cross-sectional design captures associations at a single point in time, which limits causal inference. As a result, observed relationships between job demands, job control, social support, and occupational stress should be interpreted as correlational rather than causal. Second, the use of non-probability availability sampling introduces the potential for selection bias, as nurses who were more accessible or more willing to participate may be over-represented. Certain wards, shifts, or personality characteristics may therefore be disproportionately reflected in the sample. To mitigate this limitation, data collection spanned multiple wards and shifts over 20 consecutive days to maximize variability in participant characteristics and work contexts.

Third, occupational stress was measured using a single-item, self-reported indicator, which limits the depth of construct measurement and introduces the possibility of response bias. While pragmatic for a busy clinical setting, this approach does not capture the multidimensional nature of occupational stress, and future studies would benefit from the use of validated multi-item scales. Fourth, several potentially important confounders were not measured, including individual resilience, unit-specific organizational culture, peer dynamics, and informal social support. The omission of these factors may have contributed to residual confounding, particularly in explaining some of the counterintuitive associations observed.

Notably, the inverse associations identified for inadequate staffing, lack of hospital accommodation, and gender-related stressors should not be interpreted as true protective effects. Rather, these findings likely reflect complex system dynamics, including statistical suppression, collinearity among chronic stressors, adaptation to persistently high-demand environments, and unmeasured confounding factors such as professional seniority or socioeconomic stability. Recognizing these analytical anomalies is essential to avoid misinterpretation of adjusted estimates. Additionally, the borderline events-per-variable ratio in the multivariable logistic regression models may have reduced statistical power to detect weaker associations and increased coefficient instability. This limitation was partially addressed through model parsimony and strong theoretical grounding in variable selection.

Finally, multivariable analyses were conducted using domain-specific models rather than a single unified model including predictors from all domains simultaneously. Consequently, the reported adjusted associations may be subject to residual cross-domain confounding and should not be interpreted as fully independent effects. While this domain-structured approach was retained to preserve interpretability within the JDRS framework, future research should employ unified multivariable models incorporating predictors across all domains simultaneously to estimate fully adjusted associations and confirm the robustness of the observed patterns.

Overall, while these limitations highlight areas for methodological refinement, they do not undermine the study's contributions. Instead, they underscore important directions for future research, including longitudinal designs, mixed-methods approaches, and more contextually enriched measurement strategies to further elucidate the determinants of occupational stress among nurses in resource-limited settings.

## 5 Conclusion

In conclusion, occupational stress among nurses at Mulago National Referral Hospital is highly prevalent and shaped by a convergence of extreme job demands, constrained control, and limited institutional and societal support. Framed through the JDCS model and interpreted within the broader JDRS framework, these findings suggest not only expected stress pathways but also counterintuitive patterns that reflect adaptation to chronic systemic strain. Addressing nurse stress in Uganda therefore, requires integrated interventions targeting structural capacity, workplace autonomy, and social valuation of nursing, supported by future research that captures the dynamic and contextual nature of occupational stress.

## Data Availability

The raw data supporting the conclusions of this article will be made available by the authors, without undue reservation.
